# Cytotoxicity of dental disclosing solution on gingival epithelial cells in vitro

**DOI:** 10.1002/cre2.321

**Published:** 2020-08-02

**Authors:** Im‐hee Jung, Kyeong Ho Yeon, Hwi Rin Song, Young Sun Hwang

**Affiliations:** ^1^ Department of Dental Hygiene, College of Health Science Eulji University Seongnam South Korea; ^2^ Environmental Business Team TAEYOUNG E&C Seoul South Korea

**Keywords:** apoptosis, biofilm, dental plaque

## Abstract

**Objective:**

Coloring dental biofilm and plaque with a dental disclosing solution is visually effective in dental treatment and oral hygiene education. Despite continuous reports of the risk of the product ingredients, dental disclosing solution are widely used in dentistry. However, the cytotoxic mechanism of dental disclosing solution is not known. Here we elucidated the tissue dyeing range and investigated the cytotoxic mechanism of dental disclosing solution.

**Materials and methods:**

Gingival epithelial cells and mouse head and neck tissue were stained with dental disclosing solution. Changes in the cell cycle distribution by the dental disclosing solution treatment were analyzed. A deoxynucleotidyl transferase dUTP nick and labeling (TUNEL) assay was performed to examine the apoptotic features of the gingival epithelial cells.

**Results:**

Dental disclosing solution stained the chromosome strongly, as well as both the hard and soft tissue of the mouse head and neck. The results of flow cytometric analysis and TUNEL analyses revealed that the cytotoxicity associated with dental disclosing solution was related to the induction of apoptosis. However, the staining of porcine skin by dental disclosing solution was not easily removed, even with a wide range of pH solutions.

**Conclusions:**

These results suggest that dental disclosing solution had strong cytotoxicity and safer alternatives are needed.

## 1 INTRODUCTION

Dental caries and periodontal disease are major dental diseases with very high prevalence. According to the 2017 Statistical Yearbook of Health Insurance from the Health Insurance Review and Assessment Service in Korea, the number of patients who received outpatient treatment for gingivitis and periodontal diseases (K05) was 15.18 million, ranking second in the number of outpatient diseases (Health Insurance Review and Assessment Service in Korea. Statistical Yearbook of Health Insurance, 2017). In 2017, the number of patients with gingivitis and periodontal disease doubled compared to 7.94 million in 2010. There were 5.83 million patients reported to have dental caries in 2017 compared to 5.34 million in 2010. Dental caries and periodontal disease have been noted as frequent outpatient disease for many years. The results for the global epidemiology and the global search trends of oral problems using Google Trends show that the prevalence of dental caries and severe periodontitis is also very high and steadily increasing, making it the most common disease affecting humans worldwide (Frencken et al., 2017; Patthi et al., 2017).

Dental caries is caused by the conversion of sugar to organic acids by *Streptococcus mutans* of dental plaque on the tooth surface. Organic acids, such as lactic acid, are the main cause of the decalcification of the inorganic compounds in teeth. Severe caries in infants and children leads to nursing bottle caries and ramphant caries. In patients with diabetes, low saliva efflux causes insufficient mouth washing that increase dental caries. Since a decrease in saliva secretion decreases the mouth pH, the risk of dental caries by acid resistant‐bacteria such as *Lactobacillus* and *Actinomyces* is also increased (Haque, Sartelli, & Haque, 2019). Periodontal disease is characterized by inflammatory lesions in the tissues around the teeth, such as the gingival sulcus, which are formed by the attachment of bacteria to food residue on the dental surface (Lertpimonchai, Rattanasiri, Arj‐Ong, Attia, & Thakkinstian, 2017). In particular, endotoxin such as lipopolysaccharides, exotoxin, flagella, antibiotic resistance, and proteolytic activity increase the risk of periopathogenic bacteria (Teles, Teles, Frias‐Lopez, Paster, & Haffajee, 2013). Periodontal disease is caused by a combination of factors, including bacterial growth, nutrition, innate immunity, and endocrine and systemic disease factors. The disease increases the periodontal pocket depth and degrades the periodontal ligaments and alveolar bone, leading to tooth loss. Dental plaque is a major cause of the progression of gingivitis and periodontitis, so controlling the amount of dental plaque is important to gingival health. Therefore, for the effective control and prevention of dental disease, a clear identification of the associated risk factors is important, and continuous oral hygiene management is needed.

Dental plaque is not easily observed, making it difficult to manage oral hygiene. Dental plaque disclosing solutions are dyes that color dental biofilm and dental plaque to make observation easier, thereby increasing motivation for oral hygiene and facilitating the plaque removal process. The self‐use of dental disclosing solutions has been shown to have a positive effect on oral self‐care and has been proposed as an effective method for improving oral health (Lee & Kang, 2018).

Erythrosine, known as Red No. 3, is an organo‐iodine compound and the most widely used dye in dental plaque disclosing agents. Fluorescein, which is observed under ultraviolet light, is also used as a dental plaque disclosing agent. In addition, two‐tone dye systems mixing erythrosine with Fast Green or Brilliant Blue are generally used. According to Drug Safety Country, the Pharmaceutical Integrated Information System of the Ministry of Food and Drug Safety, Red No. 3 (erythrosine) is used in dental gel, oral suspensions, hard and soft capsule agent, and other oral products in Korea. However, studies have shown that high doses cause cancer in mice and the U.S. Food and Drug Administration (FDA) partially banned erythrosine in 1990 (FDA, 1990) Chronic erythrosine ingestion was reported to cause chronic stimulation of the thyroid by thyroid‐stimulating hormone (TSH), facilitating the formation of thyroid cancer in rat (Jennings, Schwartz, Balter, Gardner, & Witorsch, 1990). A complete ban on erythrosine was implemented by the FDA, but it is still commonly used in many countries around the world, including the United States. In 1986, the cytotoxicity of dental disclosing agents was reported in cultured mammalian cells (Watanabe et al., 1986). However, there has been no reports of the tissue dyeing range and cytotoxic mechanism of dental disclosing reagents.

In this study, cultured gingival epithelial cells and experimental animal tissues were used to determine the staining range of dental disclosing solution. Also, flow cytometry analysis was performed to elucidate the cytotoxic mechanism of dental disclosing solution. This study identified the need for alternative disclosing solutions.

## 2 MATERIALS AND METHODS

2‐Tone (Blue‐Violet) used in this experiment is a popular disclosing solution in dental clinics. The solution was purchased from Young Dental Manufacturing (Earth City, MO). All chemical used in this study were of analytical grade.

Gingival epithelial cells were derived from a patient who had wisdom teeth extractions and who did not have oral mucosal disease. The cells were obtained from the Department of Oral Pathology, College of Dentistry, Yonsei University (Seoul, Korea). The gingival epithelial cells were cultured in Dulbecco's Modified Eagle Medium: Nutrient Mixture F‐12 (DMEM:F‐12, 3:1 ratio) medium with 10% fetal bovine serum (FBS) in a humidified atmosphere of 5% CO_2_ at 37°C.

To stain the gingival epithelial cells, the cells were cultured with media containing dental disclosing solution for 5 min, and staining was monitored under light microscopy (EVOS XL cell imaging system; Thermo Fisher scientific, Waltham, MA). Mouse head and neck tissue were used in the tissue staining experiments to observe soft tissue and bone tissues simultaneously. Paraffin tissue block sections were deparaffinized in xylene and rehydrated in a graded alcohol series. The tissue section was covered sufficiently with dental disclosing solution for 5 min and rinsed thoroughly with phosphate‐buffered saline (PBS) solution (pH 7.2) three times, followed by dehydration and mounting. Hematoxylin & eosin staining was also proceeding separately. The sections were mounted and images was taken using a phase‐contract microscope. For both staining experiments, the stock of dental disclosing solution was diluted 200 times with complete cell culture media (v/v).

Normal cells have the ability to recover after wounding. To observed the effect of dental disclosing solution on wound recovery capacity, a wound‐healing assay was performed. The dental disclosing solution was diluted with complete cell culture media and used to treat the cell for 24 hr. Confluent cells monolayers were wounded by scraping with a sterile pipette tip and the degree of wound recovery was monitored under light microscopy.

The 3‐(4,5‐Dimethylthiazol‐2‐yl)‐2,5‐diphenyl tetrazolium bromide (MTT) assay measures cell viability via metabolic activity. An MTT assay was performed to observe the cytotoxicity of the dental disclosing solution. Cells (5 × 10^3^ cells/well) were plated in a 96‐well culture plate in complete cell culture media and incubated overnight to adhere. Then, the cells were incubated with diluted media containing dental disclosing solution for 24 hr. The cells were incubated with a 5 mg/ml MTT solution for an additional 1 h at 37°C. The formazan product was dissolved in 200 μl dimethyl sulfoxide (DMSO) and the absorbance was measured at 570 nm in a microplate reader (Synergy™ HTX Multi‐Mode Microplate Reader; BioTek Instruments, Inc., Winooski, VT).

Staining with the dental disclosing solution and decolorization were tested in porcine skin. Porcine skin is widely used in percutaneous phenotyping research to substitute for human skin (Jacobi et al., 2007). All decolorizing solution (drinking vinegar, orange juice, Listerine, milk, distilled water, green tea, Pocari Sweat, bottled water, toothpaste, and alkaline water) were used immediately after opening. Dental disclosing solution was dropped onto porcine skin and left for 5 min. After absorbing the excess solution with a tissue, the skin was covered with various solutions for decolorizing and left it for 30 min with agitation. The degree of decolorization was analyzed by ImageJ program (National Institutes of Health).

Cell cycle analysis by measuring the DNA content is a method that most frequently employs flow cytometry to distinguish cells in different phases of the cell cycle. The cells were cultured with diluted media containing dental disclosing solution for 24 hr. Then, the cells were collected and fixed in 70% ethanol. The cells were incubated with 40 μg/ml of propidium iodide (PI) and 200 μg/ml RNase for DNA content analysis. Fluorescence was measured with a FACScan Flow Cytometer (Becton Dickinson, San Jose, CA) and analyzed using CellQuest and Modfit LT programs (Becton Dickinson).

Normal epithelial cells maintain intercellular attachment, but the loss of intercellular attachment and DNA fragmentation is observed when apoptosis is induced. Terminal deoxynucleotidyl transferase dUTP nick end labeling (TUNEL) is a method for detecting DNA fragmentation by labeling the 3′‐hydroxyl termini in the double‐strand DNA breaks generated during apoptosis. Cells (1 × 10^3^ cells/well) were incubated for 24 hr in culture medium with dental disclosing solution and washed twice with PBS, followed by fixing in 4% paraformaldehyde and permeabilizing in Triton‐X100 solution. Alexa Fluor 647 phalloidin was added for 4 hr to stain actin. Cell morphology was observed under a fluorescence microscope (EVOS FL Cell Imaging System, Thermo Fisher Scientific). The TUNNEL assay was conducted using an In Situ Cell Death Detection Kit (Roche Diagnostics, IN) according to the manufacturer's instruction. 4′,6‐Diamidino‐2‐Phenylindole (DAPI) was used to stain the DNA.

Statistical analyses were conducted using InStat GraphPad Prism ver. 5.01 statistical software (GraphPad Software, Inc., San Diego, CA). The results are expressed as the means ± standard deviation (*SD*). The experiments were repeated three times and representative results are shown for each experiment. The statistical significance of the differences between the groups was analyzed via repeated measures of one‐way analysis of variance and Tukey's post‐hoc analysis. The non‐parametric Mann–Whitney test was used to evaluate the results of the TUNEL assay. *p*‐Values of <.05 were considered statistically significant.

## 3 RESULTS

**FIGURE 1:**
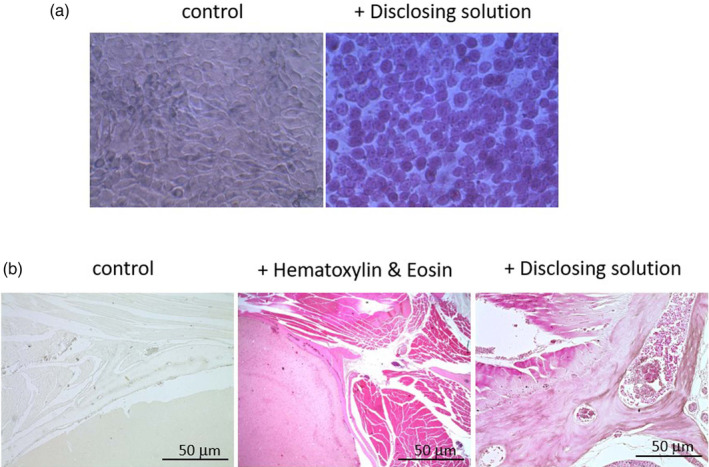
Gingival epithelial cells and tissue staining with dental disclosing solution. (a) Gingival epithelial cells were treated for 5 min with the dental disclosing solution (1/200 dilution in complete media, v/v) and staining was monitored under light microscopy. (b) Deparaffinized mouse head and neck tissue were stained with hematoxylin & eosin (H&E) or dental disclosing solution (40× magnification). The sections were mounted and images were taken using a phase‐contract microscope. (Scale bar 50 μm)

To observe the range of tissues stained by the dental disclosing solution, cultured gingival epithelial cells, and deparaffinized mouse head and neck tissue were stained with disclosing solution. As shown in Figure 1a, strong chromosomal staining of the nuclei was observed, as well as cytoplasmic staining in the gingival epithelial cells. A wide variety of mouse head and neck tissue, including skeletal muscle, collagenous fibers, fatty marrow, cellular hematopoietic tissue, and cortical bone, were stained with dental disclosing solution. The extent and degree of staining by disclosing solution were similar to that of hematoxylin & eosin (H&E) staining (Figure 1b).

**FIGURE 2:**
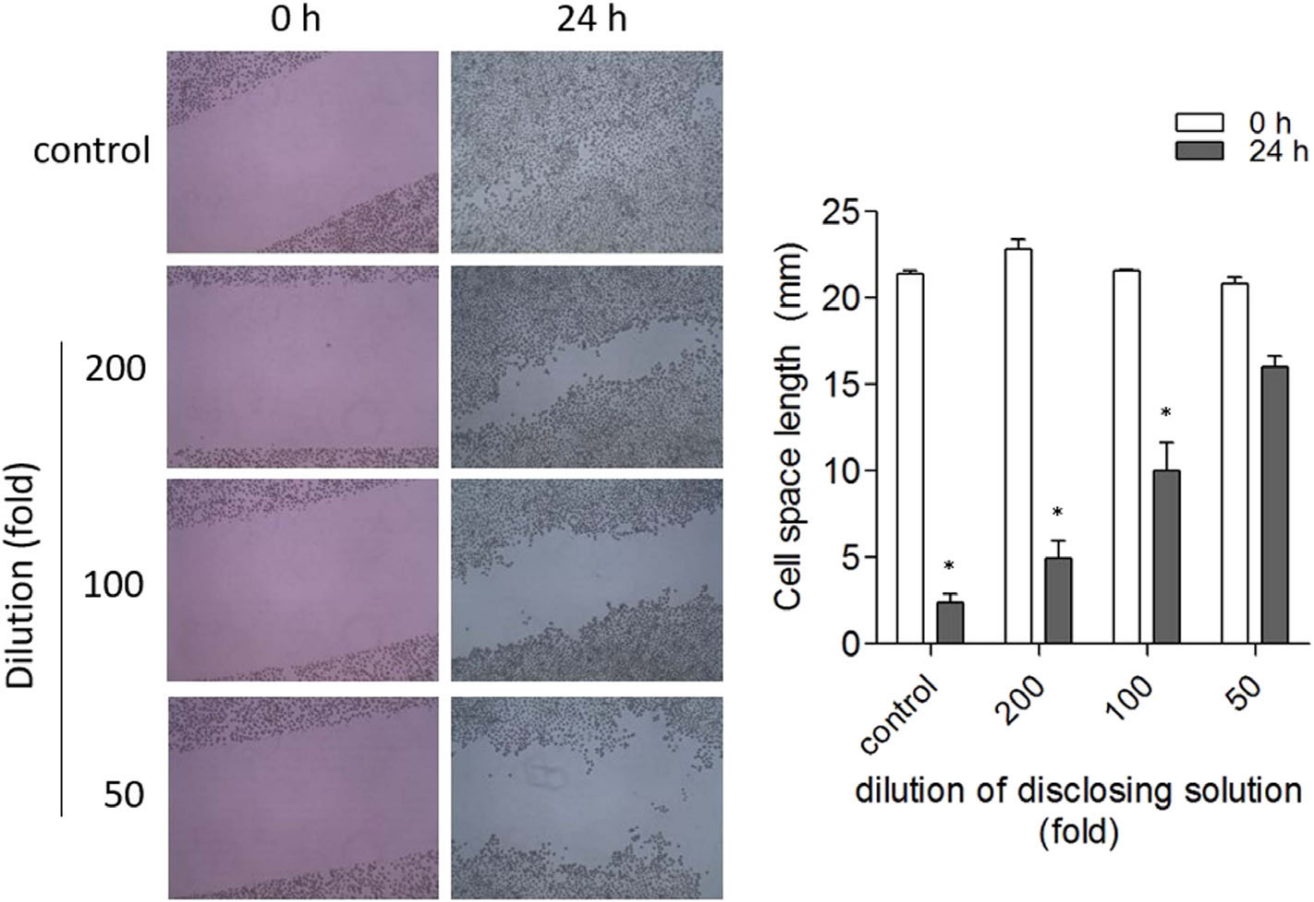
Effect of dental disclosing solution on wound healing. Confluent cell monolayers were scraped with a sterile pipette tip in the presence or absence of dental disclosing solution and cells that migrated into the wounded monolayer were captured after 24 hr. The values of individual experiments are expressed as the mean ± standard error of three independent experiments. *p < .001 versus control

Wound healing is the physiological process of injury repair. Delayed wound recovery leads to infection vulnerability and non‐healing chronic wounds (Guo & Dipietro, 2010). To test the effect of dental disclosing solution on wound recovery, confluent gingival epithelial cell monolayers were wounded by scraping with a sterile pipette tip and wound recovery of the surrounding cells was observed. As shown in Figure 2, the wound was completed recovered in the control cells. However, wound healing in media containing dental disclosing solution was delayed in a concentration‐dependent manner. Wound healing failure was observed in media diluted 50‐fold with dental disclosing solution.

**FIGURE 3:**
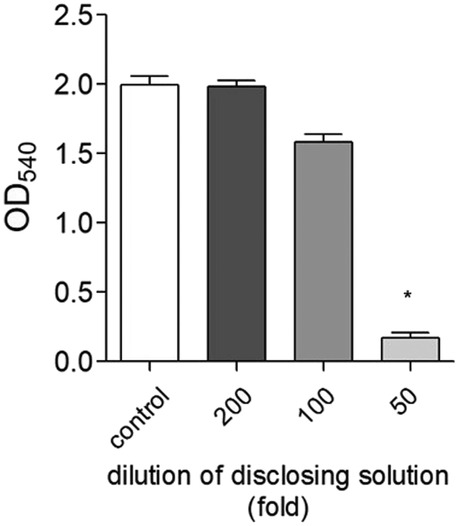
Effect of dental disclosing solution on cell viability. The cells were cultured in the presence or absence of dental disclosing solution at the indicated dilutions for 24 hr and an MTT assay was performed. The values of the individual experiments are expressed as the mean ± standard error of three independent experiments. *p < .001 versus control

To determine the reason for the lack of wound healing, the number of viable cells was estimated using the MTT assay. In media diluted 200‐ or 100‐fold with dental disclosing solution (v/v), the cell survival was similar to the control cells, whereas a significant reduction in cell survival was onserved in media diluted 50‐fold with dental disclosing solution (Figure 3).

**FIGURE 4:**
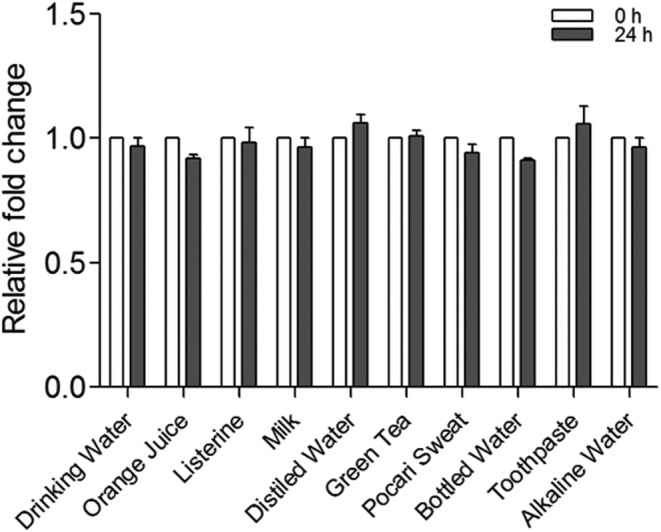
Tissue destaining of the dental disclosing solution. Porcine skin was stained with dental disclosing solution for 5 min and the remaining solution was absorbed with tissue paper. Then destaining was observed using solutions of varying pH values. The images were acquired and captured in Image J (National Institutes of Health) for density quantification, and calculated as pixel intensities. The values of the individual experiments are expressed as the mean ± standard error of three independent experiments. Representative results are shown for each experiment. No significant change was observed compared to the 0 hr control of each solution (*p* > .05)

To identify a solution that could bleach dental disclosant staining easily, disclosing solution was dropped on porcine skin and absorbed extra solution after 5 min. Then, the stained area was covered with solutions with a variety of pHs, including drinking vinegar, orange juice, Listerine, milk, distilled water, green tea, Pocari Sweat, bottled water, toothpaste, and alkaline water. As shown in Figure 4, no destaining solution bleached the disclosant stain any more than the distilled water control.

**FIGURE 5:**
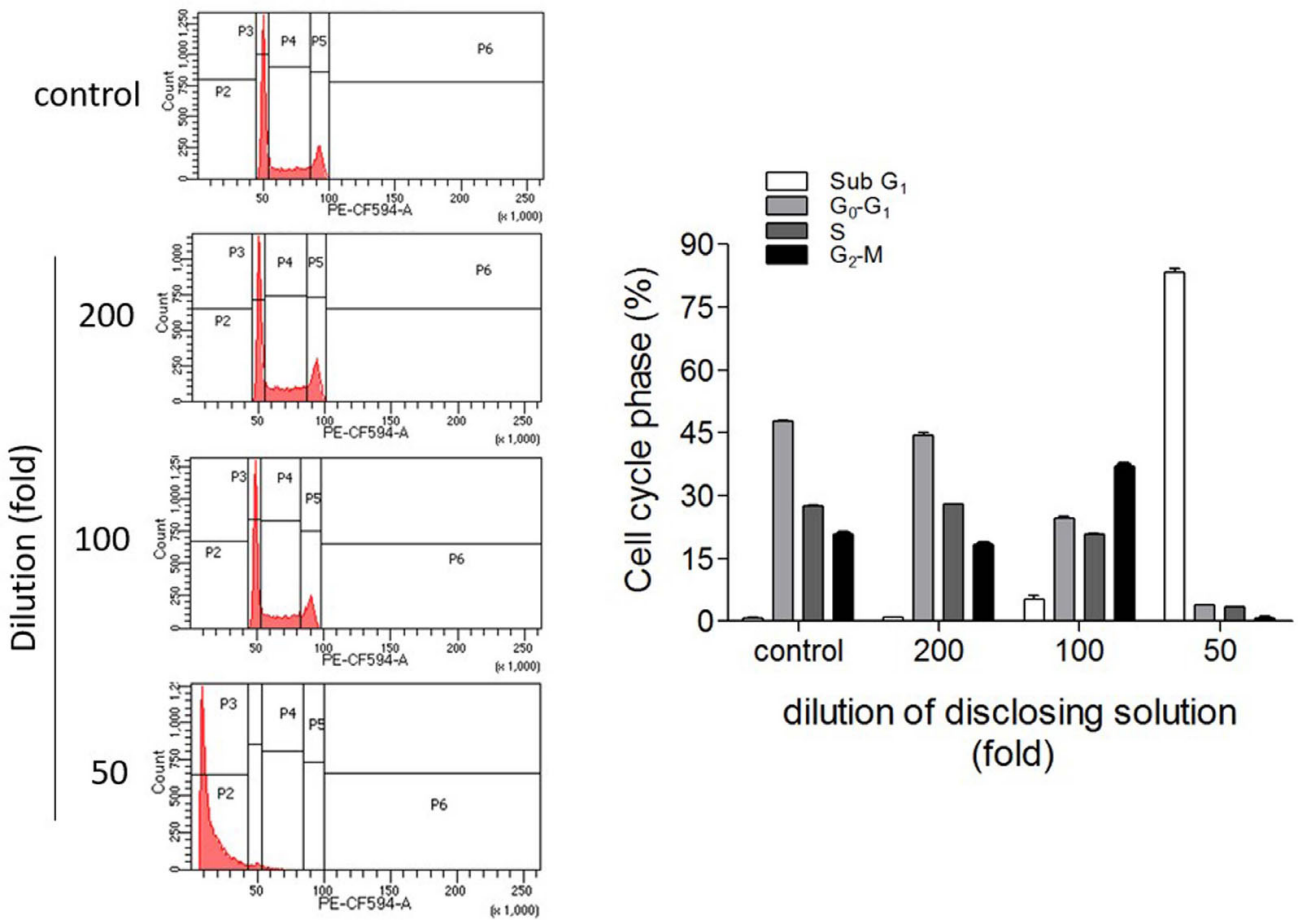
Effect of dental disclosing solution on cell cycle distribution. The cells were cultured in the presence or absence of dental disclosing solution at the indicated dilutions 24 hr. Then, the cells were fixed, the nuclei were stained with propidium iodide (PI), and the cell cycle distribution was analyzed by flow cytometry. The percentage of cells in each cell cycle phase of each experimental group was graphed

To elucidate the cytotoxic mechanism of dental plaque disclosing solution, we assessed the cell cycle distribution in gingival epithelial cells treated with varying dilutions of dental disclosing solution (Figure 5). The results of flow cytometric analysis showed that the cell cycle distribution in cells in media diluted 200‐fold with dental disclosing solution (v/v) was similar to the control cells. However, those in media diluted 100‐fold with dental disclosing solution were changed. The proportion of Sub G_1_ cells were significantly increased in cells in media diluted 50‐fold with dental disclosing solution.

**FIGURE 6:**
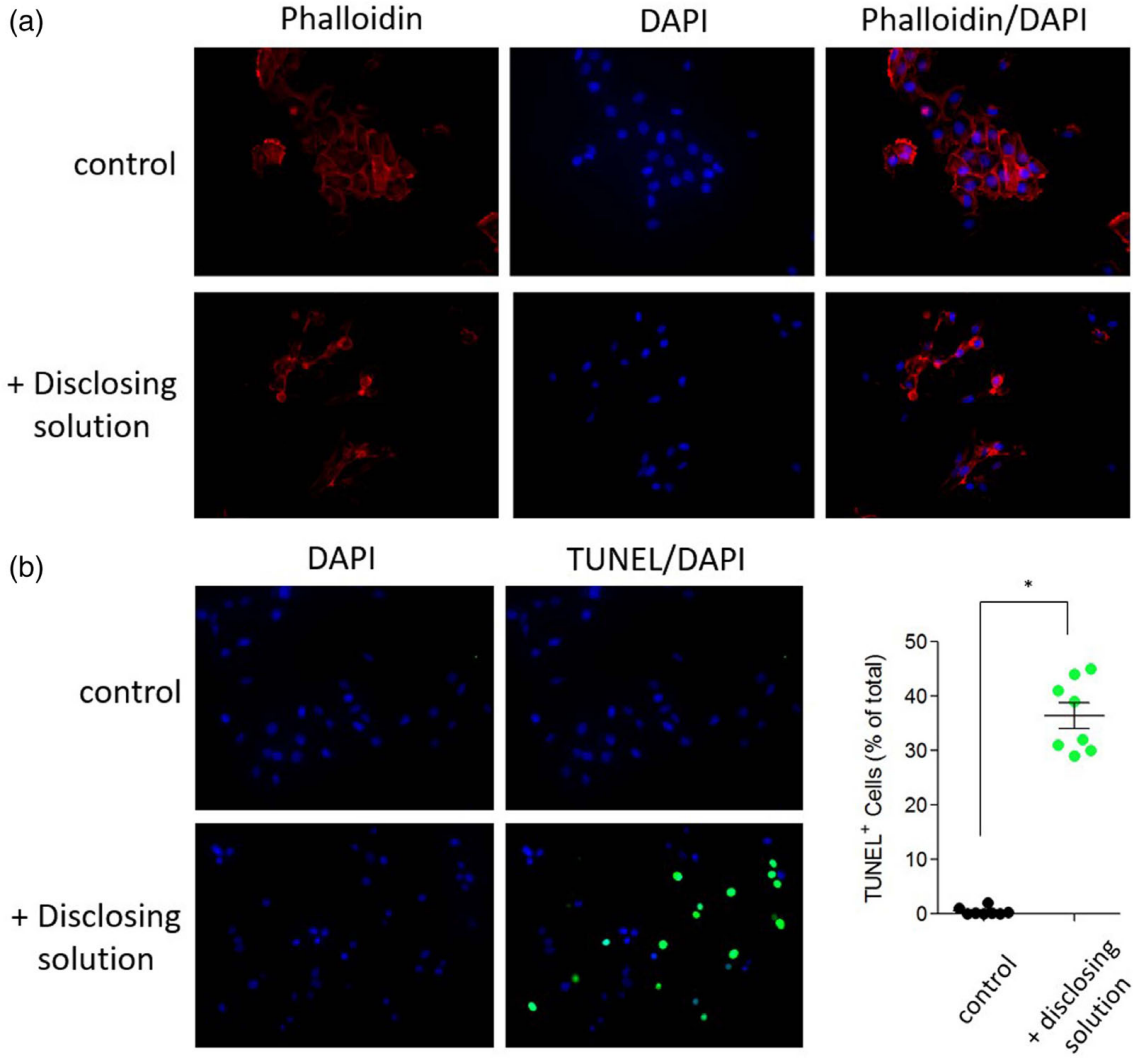
Effect of dental disclosing solution on cell morphology and cell death. (a) The cells were cultured in the presence or absence of dental disclosing solution (50‐fold dilution) and stained with Alexa Fluor 647 phalloidin and 1 μg/ml DAPI. (b) A TUNEL assay was performed on the dental disclosing solution (50‐fold dilution)‐treated cells using an In Situ Cell Death Detection Kit. Intensely stained TUNEL‐positive cells (green) were visualized under a fluorescence microscope (40× magnification). The results were normalized to the total number of DAPI^+^ cells. *p < .001 versus control. Representative images are shown

The effect of the dental disclosing solution on cell morphology was observed by staining the cellular structure actin protein with a fluorescent dye. The morphology of the control cells, in which the intercellular attachment was maintained, was round (Figure 6a). However, cells in the culture media containing dental disclosing solution lost intercellular adhesion ability and were spindly and irregularly shaped. TUNEL is a method for detecting DNA fragmentation by labeling the 3′‐hydroxyl termini in double‐strand DNA breaks generated during apoptosis (Gorczyca, Traganos, Jesionowska, & Darzynkiewicz, 1993). A higher TUNEL signal was observed in the cells cultured in the disclosing solution media than in the control cells and the location of the fluorescence was the same as that of the nucleic acid DAPI staining (Figure 6b).

## 4 DISCUSSION

Studies have shown that oral health education using dental disclosing solution is effective in preventing dental caries and periodontitis (Fasoulas et al., 2019; Lee, Jung, & Pang, 2016). The primary cause of dental caries and periodontitis is the attachment of dental plaque to dental fissures and gingival sulcus. Dental plaque is a bacterial mucosa that forms on the surface of teeth, gingiva, and other oral structures and is a host‐dependent biofilm. Therefore, prevention is more important than the treatment of oral disease and oral disease can be prevented though oral hygiene management. The management of dental plaque can be prevented by self‐care and expert care. However, full management by experts is not possible because dental plaque is continuously produced. Regular visits to the dentist and scaling of areas where brushing missed are usually effective ways to manage dental plaque. However, busy people have problems fitting in dental visits and long‐term brushing is effective, but bad brushing results in poor oral conditions. Teaching proper brushing techniques should be done through oral health education and individual education is more effective than group education (Sheiham, 1979). In individual education, the dental disclosant method is more effective in managing oral hygiene than regular brushing education because it can maximize the visual effects and motivate better oral hygiene (Lee, Jung, & Pang, 2016).

Dental disclosants penetrate dental plaque and dye the entire plaque, making it easier to identify bacterial plaque. Dental disclosants are available in liquid, tablet, lozenge, and gel forms. The liquid type can be applied to the affected area and bacterial plaque is identified after washing with water. Compared with the red color of bacterial plaque, longer accumulated bacterial plaque is dyed blue. Bacterial plaque with very low pH is dyed light blue. When tablet‐type dental disclosants are applied to the teeth, the tongue is used to melt them and unnecessary dye spreads throughout the mouth, including the tongue, and the dyed soft tissues are not easily discolored. In this study, experiments using mouse head neck tissue showed extensive soft tissue and bone tissue staining by the dental disclosing solution, which was not easy to remove, even with absolute alcohol. In addition, strong cytotoxicity to oral epithelial cells was observed in the MTT assay. Through cell cycle distribution analysis and the TUNEL assay, the cytotoxic mechanism of the dental disclosing solution was determined to be apoptosis. Discoloration of the dental disclosing solution was not easy, either by drinkable items with various pHs or by oral cleansing agents, as shown in Figure 4. Therefore, reconsideration of the use of dental disclosants is urgently necessary.

The major ingredients of dental disclosants are erythrosine (Red No. 3) and Fast Green (Green No. 3). Coloring agents are widely used in various forms, including oral and external agents, and in the food, cosmetics, and dyeing industries. Regulations on coloring agents differ from country to country. Although the U.S. FDA banned the use of amaranth (Red No. 2) in 1976, the Korean Food and Drug Administration (KFDA) requested only a user restriction in 2004 and did not ban the coloring agent. Among the food coloring permitted in Korea, erythrosine is a type of tar‐based pigments, which is widely used in dyeing fabrics, as well as in food (Food & Drug Administration, 2015). Erythrosine is rarely used in the United States because of its toxicity. Erythrosine causes serotonin secretion disorder, which affects the autonomic nervous system, causing problems such as depression and personality disorders (Yankell & Loux, 1977). Tar‐based pigments, such as erythrosine have a low lethal dose (LD50) of 6.7–7.4 g/kg when they are ingested as a single ingredient, but unexpected hazards, such as teratogenic effects from drinking water containing tar‐based pigments, have been reported (Collins, Black, O'Donnell Jr, Shackelford, & Bulhack, 1993). In addition, iodine, a component of erythrosine, is absorbed into the human body during long‐term intake, causing thyroid cancer and liver cancer. It is not safe to exceed the safety limits set forth by the KFDA or the World Health Organization (WHO). However, infants, seniors, patients with weak immune systems, and atopic patients should choose products that fewer or no additives.

The Ministry of Health and Welfare designates and manages chemical compounds that are manufactured, processed, imported, used, stored, or displayed for sale, only if they are not believed to be harmful to human health. In Korea, 370 kinds of chemical compounds and 50 kinds of assistive agents are permitted as food additives. Among the food tar pigments currently allowed in Korea, Red No. 2 and Red No. 102 are prohibited in the United States, whereas Green No. 3 is permitted in the United States and Japan, but not in the EU. In the notice No. 87‐42 of the Ministry of Health and Social Affairs in July 1987, the government designated tar pigments for medicine, non‐medicine products, and cosmetics among organic tar pigments and prescribed the standards and testing methods. A total of 76 kinds of tar pigments were classified for external use in May 2009 after a number of revisions and were designated as tar pigments for use in medical products, non‐medical products, and cosmetics (Ministry of Food and Drug Safety, 2014).

Fluorescein and erythrosine were reported to display antibacterial effects against *Streptococcus mutans* (Wood, Metcalf, Devine, & Robinson, 2006). In addition, dental disclosing solution is very effective for oral hygiene education. However, considering that dental disclosing solution is highly cytotoxic to oral epithelium cells and difficult to bleach, studies on easy decolorizing methods are needed. To effectively remove erythrosine from food and dyeing wastewater, adsorption using chicken feathers and adsorption removal methods using activated carbon have been investigated (Gupta, Mittal, Kurup, & Mittal, 2006; Lee & Yoon, 2009). An activated carbon adsorption process has been used for dye removal and metal ion adsorption for drinkable water and a various other purpose (Foo & Hameed, 2010; Lee et al., 2016). Product development using activated carbon will be useful for oral hygiene management.

It is also necessary to investigate alternative dental disclosants with good staining ability and biocompatibility. The use of dental disclosing solution should be conducted under the guidance of an expert and care must be taken not to stain areas outside of the teeth as much as possible. While the risk of dental disclosants may be mitigated by the cleansing action of saliva in the mouth, patients who have oral inflammation, oral dryness, or poor oral hygiene should limit its use.

In conclusion, this study found that the dental disclosing solution stained soft tissue and bone tissue broadly and was not easy to remove after dyeing. The dental disclosing solution induced apoptosis, indicating strong cytotoxicity. Based on these results, we suggest that the development of an alternative disclosing solution is urgently needed.

## CONFLICT OF INTEREST

The authors have no conflict of interest to disclose.
